# 

**Published:** 1816-10

**Authors:** 


### TO CORRESPONDENTS.

The Reviewer of Dr. Parry's Work on the Arterial Pulse, has on hand a train of experiments to illustrate the Circulation of the Blood; which, with a Plate of the Apparatus, will be given in our next, together with Dr. Parry's Reply to the Reviewer.

Dr. Johnson on Syncope Angens, will appear in our November Number.

Mr. Shoveller, on the Fever which occurred in the Montague, at Gibraltar, will be inserted as early as possible.

The interesting Cases and Observations, sent by Mauritius Power, Esq. have been received; his Communications will ever be esteemed.

Mr. Norman's favour came safely to hand.

We regret that we have been obliged to omit, in consequence of a pressure of Review Matter, Morgagni's Morbid Anatomy; it will, however, for the future, be continued in regular succession.

Review of Lambe's Additional Reports; Mr. Jardine's Work on the Improvement of Surgical Instruments; and Reid on Nervous Affections, we shall endeavour to insert in our ensuing publication.

*We have received several letters from the Country, stating, that all applications for our Journal were vain, as the answer was, that no Journal of the kind was in existence! and some other Work was always sent in its stead. Such despicable schemes to obstruct the fair exertions of industry and independence will not long succeed, and may ultimately recoil on the heads of the delinquents themselves.*

☞ Authors and Publishers are requested to send Copies of new Works for Review as early as possible after their publication.

